# New Role of Adult Lung c-kit^+^ Cells in a Mouse Model of Airway Hyperresponsiveness

**DOI:** 10.1155/2016/3917471

**Published:** 2016-12-20

**Authors:** Giuseppe Spaziano, Donato Cappetta, Konrad Urbanek, Elena Piegari, Grazia Esposito, Maria Matteis, Manuela Sgambato, Gioia Tartaglione, Rosa Russo, Raffaele De Palma, Francesco Rossi, Antonella De Angelis, Bruno D'Agostino

**Affiliations:** ^1^Department of Experimental Medicine, Section of Pharmacology, Second University of Naples, 80138 Naples, Italy; ^2^Department of Clinical and Experimental Medicine, Second University of Naples, 80137 Naples, Italy

## Abstract

Structural changes contribute to airway hyperresponsiveness and airflow obstruction in asthma. Emerging evidence points to the involvement of c-kit^+^ cells in lung homeostasis, although their potential role in asthma is unknown. Our aim was to isolate c-kit^+^ cells from normal mouse lungs and to test whether these cells can interfere with hallmarks of asthma in an animal model. Adult mouse GFP-tagged c-kit^+^ cells, intratracheally delivered in the ovalbumin-induced airway hyperresponsiveness, positively affected airway remodeling and improved airway function. In bronchoalveolar lavage fluid of cell-treated animals, a reduction in the number of inflammatory cells and in IL-4, IL-5, and IL-13 release, along with an increase of IL-10, was observed. In MSC-treated mice, the macrophage polarization to M2-like subset may explain, at least in part, the increment in the level of anti-inflammatory cytokine IL-10. After in vitro stimulation of c-kit^+^ cells with proinflammatory cytokines, the indoleamine 2,3-dioxygenase and TGF*β* were upregulated. These data, together with the increased apoptosis of inflammatory cells in vivo, indicate that c-kit^+^ cells downregulate immune response in asthma by influencing local environment, possibly by cell-to-cell contact combined to paracrine action. In conclusion, intratracheally administered c-kit^+^ cells reduce inflammation, positively modulate airway remodeling, and improve function. These data document previously unrecognized properties of c-kit^+^ cells, able to impede pathophysiological features of experimental airway hyperresponsiveness.

## 1. Introduction

Asthma is a chronic inflammatory disorder of the airways characterized by variable airway hyperresponsiveness (AHR) and obstruction. It is estimated that about 300 million people worldwide suffer from asthma and that globally asthma accounts for about one in every 250 deaths [1–3]. The establishment of chronic inflammation is at the basis of mucus overproduction and airway remodeling that result in bronchial hyperactivity and variable degree of airflow obstruction [4–6]. In this regard, the release of several growth factors and the activation of local progenitor cells are important aspects to control inflammation and airway remodeling thus preventing asthma exacerbation [7, 8].

Regionally distinct resident cells with stem/progenitor characteristics include basal cells and their side population [9, 10], Clara cells and their variants [11], bronchoalveolar stem cells [12], alveolar epithelial type II progenitor cells [13, 14], lung-derived mesenchymal stem cells (MSCs) [15], and c-kit^+^ stem cells [16–19].

The importance of c-kit^+^ cells in lung homeostasis has been emphasized by the observations that c-kit mutant mice show abnormal lung architecture and that the expansion of epithelial progenitors depends on c-kit activation [20]. The contribution of c-kit receptor (also known as CD117 or stem cell factor receptor) during lung development has been demonstrated in newborn genetically modified mice whose lungs show a massive contribution of c-kit^+^ cells [21]. Furthermore, c-kit receptor has been detected on CD133^+^ epithelial progenitor cells [22] and in endothelial cells of alveolar capillaries [23]. A recent paper has tested the lineage potential of c-kit^+^ cells, evidencing their direct contribution to the vascular endothelial cell fate [17], whereas another study conducted on human fetal and postnatal lungs has claimed that c-kit expression marks a progenitor population restricted to endothelial lineage, suggesting a potential involvement of c-kit signaling in lung vascular development [18]. Finally, in an experimental model of lung emphysema, c-kit-expressing cells mitigate the progression of the disease upon being activated by hepatocyte growth factor [24].

Although the presence of c-kit^+^ cells in the lung has been repeatedly reported, suggesting that this receptor (and its endogenous ligand) may have clinical significance, properties of c-kit-bearing cells have not been completely elucidated. Therefore, the objective of this study was to test which role c-kit^+^ cells, isolated from normal mouse lungs, may play in inflammatory processes and airway remodeling that underlie pathophysiology of AHR in an animal model.

## 2. Methods

### 2.1. Cell Isolation and Culture

 Six/eight lungs were harvested from 2-3-month-old BALB/c male mice (Harlan Laboratories, San Pietro al Natisone, Italy) for each isolation of murine lung c-kit^+^ cells and fibroblasts. Samples were collected in 100 mm diameter culture dishes and were quickly washed with DPBS w/o Ca^2+^ and Mg^2+^ (Euroclone, Milan, Italy) to wash out blood. Large vascular and bronchial components were removed as well. In order to obtain a cell suspension, lungs were tiny minced and enzymatically dissociated with a prewarmed collagenase solution [280 U/mL type II collagenase (Worthington, Lakewood, NJ, USA); 100 U/mL penicillin and 100 mg/mL streptomycin (pen/strep, Euroclone)]. After a 45 min digestion at 37°C under agitation, collagenase was inactivated by adding a double volume of precooled quenching buffer [0.5% bovine serum albumin (Sigma-Aldrich, St. Louis, MO, USA); pen/strep]. Cell suspension was further purified by several passages through cell strainers [70 and 40 *μ*m pores (BD Biosciences, Franklin Lakes, NJ, USA)] and centrifuged at 1200 rpm for 10 min to remove debris. Cell pellet was collected and then washed with DPBS. After centrifugation (1200 rpm for 10 min), cell pellet was plated in 60 mm diameter culture dishes. Cells were cultured in Ham's F12 medium (Euroclone), supplemented with 10% (v/v) fetal bovine serum (Thermo Fisher Scientific, Waltham, MA, USA), 5% (v/v) horse serum (Peprotech, Rocky Hill, NJ, USA), 100 mM glutathione (Sigma-Aldrich), 10 U/mL erythropoietin (Sigma-Aldrich), 100 *μ*g/mL bFGF (Peprotech), and pen/strep. Twenty-four hours later, floating cells were removed and adherent cells, mainly composed of fibroblasts, were cultured under the same conditions. Supernatant was saved, centrifuged (1200 rpm for 10 min), and plated separately. Supernatant cell cultures were expanded until P2 to undergo immunomagnetic cell sorting in order to obtain c-kit^+^ cells. Specifically, mouse CD117 microbeads were employed according to manufacturer's instructions (Miltenyi Biotec, Gladbach, Germany). At P2-P3, cells were characterized by FACS and immunolabeling to assess cell phenotype.

### 2.2. FACS Analysis

Total isolated cells extracted from lung tissue were analyzed by FACS (FACScalibur, BD Biosciences) for the detection of a c-kit^+^ subpopulation (BD Biosciences, Italy). After magnetic sorting, c-kit-enriched cells were analyzed for hematopoietic lineage marker by using CD45 antibody (BD Biosciences). Isotype-matched negative controls were utilized to define the threshold for each specific signal and to establish the appropriate gating. Data were analyzed with the instrument software.

### 2.3. In Vitro Stimulation of c-kit^+^ Cells

1.5 × 10^5^ c-kit^+^ cells were simultaneously stimulated with 10 ng/mL TNF*α* and 10 ng/mL IFN*γ* (Merck Millipore, Darmstadt, Germany) to mimic inflammatory environment [25]. Total RNA was extracted after 3, 6, 12, and 24 h. IFN*γ* and TNF*α*, commonly used cytokines for in vitro cell priming, induce secretion of molecules involved in the regulation of tissue homeostasis, including indoleamine 2,3-dioxygenase (IDO) and prostaglandin E2 [26].

### 2.4. Quantitative RT-PCR

Total RNA was extracted with TRIzol from rested and primed c-kit^+^ cells for the detection of IDO, transforming growth factor *β* (TGF*β*), and IL-10 transcripts (KiCqStart SYBR Green Primers; Sigma-Aldrich). HPRT was used as housekeeping gene. iScript One-Step RT-PCR Kit with SYBR Green (Bio-Rad Laboratories, Milan, Italy) was employed to perform quantitative RT-PCR and 3 ng of total mRNA from any sample was used as template. Cycling conditions were performed according to manufacturer's instructions: cDNA synthesis (10 min at 50°C); reverse transcriptase inactivation (5 min at 95°C); PCR cycling and detection (42 cycles; 10 sec at 95°C; 30 sec at 58°C); melt curve analysis (1 min at 95°C, 1 min at 55°C, 5 sec at 55–95°C, increasing by 0.5°C each cycle). A CFX96 Real-Time PCR Detection System was employed (Bio-Rad Laboratories).

### 2.5. Immunocytochemistry

For immunolabeling, c-kit^+^ cells were fixed in 4% paraformaldehyde and incubated with anti-CD117 (Santa Cruz Biotechnology, Dallas, TX, USA). Omission of the primary antibody was used as a negative control.

### 2.6. GFP Lentiviral Infection

After in vitro expansion, c-kit^+^ cells were infected with Cignal Lentivirus carrying GFP and puromycin resistance genes at a MOI of 50 (Qiagen, Milan, Italy). After 24 h, cells were washed and infection medium was replaced by fresh medium. At this time, Cignal reporter constructs were integrated into the genomic DNA. To select the cells stably expressing the reporter GFP gene, puromycin (5 *μ*g/mL) selection was performed. GFP^+^ c-kit^+^ cells were collected by centrifugation and diluted at the density of 5 × 10^4^ cells/50 *μ*L in the appropriate medium and used for in vivo procedures.

### 2.7. Experimental Protocol

To induce asthma, BALB/c mice (Harlan Laboratories) at 6 weeks of age were sensitized by s.c. injections of 0.4 mL of 10 *μ*g OVA, absorbed to 3.3 mg of aluminum hydroxide gel in sterile saline at days 0 and 7. From day 21, mice were challenged with nebulized OVA (1% in PBS) for 7 min, three days per week for three weeks by an ultrasonic nebulizer (De Vilbiss Health Care, Tipton, UK). OVA derived from chicken egg is a frequently used allergen that induces an allergic pulmonary inflammation in laboratory rodents [27, 28]. Mice were randomized into three experimental groups: (1) control group (*n* = 13), not subjected to any sensitization and treatment; (2) OVA group (*n* = 16), sensitized and challenged with OVA and injected with medium; (3) OVA+c-kit^+^ cells (OVA+cCs) group (*n* = 20), sensitized and challenged with OVA and treated with c-kit^+^ cells (5 × 10^4^ cells/50 *μ*L medium). Either medium or c-kit^+^ cells were intratracheally administered on day 31, 24 h after the second week of OVA challenge. All mice were sacrificed 10 days later and lung reactivity test or bronchoalveolar lavage (BAL) was performed. Separate sets of animals were used for lung reactivity assay or BAL collection to avoid the possibility that BAL procedure could affect lung reactivity measurements. After the assessment of lung reactivity, lungs were perfused and fixed with 10% phosphate-buffered formalin for histology. A schematic representation of the experimental protocol is displayed in Figure 2(a). Additional animals (*n* = 5) were given lung fibroblasts (5 × 10^4^ cells/50 *μ*L medium) to perform a comparative analysis of lung function.

### 2.8. Intratracheal Cell Administration

Prior to cell administration, mice were anesthetized with ketamine HCl (40 mg/kg, i.p.) and medetomidine HCl (0.15 mg/kg, i.p.). A 20-gauge custom-made catheter was inserted into the trachea via the mouth and connected to a mouse ventilator (Harvard Apparatus, Holliston, MA, USA). After confirming the correct position of the catheter in the trachea and disconnecting the ventilator, c-kit^+^ cells or lung fibroblasts were delivered through the catheter using a syringe with a fine needle. Afterward, mice were mechanically ventilated for 3 min and then placed in a warm chamber until they recovered consciousness, usually within 5–15 min. Mice from the OVA group received equal volume of medium.

### 2.9. Lung Reactivity Assay

Lung reactivity was assessed in isolated and perfused mouse lung model. As previously described, water-jacketed perspex chamber (water temperature, 37°C) was used to accommodate surgery, perfusion, and ventilation [29]. In anesthetized mouse, the trachea was exposed and cannulated. The abdomen was opened, the diaphragm was cut, and 50 *μ*L of heparin were injected into the heart. The mouse was exsanguinated after the incision of the renal vein, the thorax was opened, and the two thoracic halves were fixed with two cannulae at sides on the cork plate. Afterward, pulmonary artery was cannulated through and fixed by the ligature. Increased concentrations of acetylcholine (ACh) were administered through the arterial cannula into the pulmonary artery. The lungs were perfused in a nonrecirculating fashion through the pulmonary artery at a constant flow of 1 mL/min resulting in a pulmonary artery pressure of 2-3 cm H_2_O. As a perfusion medium, RPMI 1640 lacking phenol red (37°C) that contained 4% low endotoxin grade albumin was used. The lungs were ventilated by negative pressure (−3 to −9 cm H_2_O) with 90 breaths/min and a tidal volume of about 200 *μ*L. Every 5 min a hyperinflation (−20 cm H_2_O) was performed. Artificial thorax chamber pressure was measured with a differential pressure transducer (Validyne DP 45–24, Validyne Engineering, Northridge, CA, USA), and airflow velocity with a pneumotachograph tube connected to the differential pressure transducer. The lungs respired humidified air. The arterial pressure was continuously monitored with a pressure transducer (Isotec; Healthdyne Cardiovascular, Costa Mesa, CA, USA) which was connected with the cannula ending in the pulmonary artery. All data were transmitted to a computer and analyzed by the Pulmodyn software (Harvard Apparatus). For lung mechanics, the data were analyzed by applying the following formula: *P* = *V* · *C* − 1 + *RL* · *dV* · *dt* − 1, where *P* is chamber pressure, *C* pulmonary compliance, *V* tidal volume, and *RL* airway resistance. After 60 min, the mean tidal volume, mean airway resistance, and mean pulmonary artery pressure were stabilized at the baseline. The measured airway resistance was corrected for the resistance of the pneumotachometer and the tracheal cannula of 0.6 cm H_2_O s/mL. Successively, a repetitive dose-response curve to ACh (10^−8^ to 10^−3 ^M) in all experimental groups was obtained.

### 2.10. Bronchoalveolar Lavage

BAL was collected as follows: 0.5 mL of saline was instilled and withdrawn from the lungs via an intratracheal cannula. Lavage was repeated three times, and different samples were collected. BAL was centrifuged at 1000*g* for 10 min at 4°C. The supernatant was transferred into tubes and stored at −70°C for cytokine analysis. Cell pellets were resuspended in phosphate-buffered saline to a final volume of 0.5 mL for total and differential cell count.

### 2.11. Total and Differential Cell Count

Total cell count was performed with the Countess automated cell counter (Thermo Fisher Scientific) which evaluates cell count and viability using trypan blue stain according to the manufacturer's instructions. Differential counting was performed by Reastain Diff-Quik kit (Thermo Fisher Scientific) and at least 300 cells were counted on each cytospin according to standard morphologic criteria under light microscopy.

### 2.12. Cytokine Assay

Measurement of cytokines in the BAL was performed taking advantage of a well-established method, Luminex® xMAP technology, that allows measuring a panel of multiple analytes on a small volume sample (100 *μ*L) simultaneously [30]. The assay, for the quantitative detection of IL-4, IL-5, IL-10, and IL-13, was performed using a Milliplex Cytokine Panel plate (Merck Millipore) according to the manufacturer's instructions on automated immunoassay analyzer (Luminex 200™ System, Thermo Fisher Scientific) as previously described [31]. All samples were run in duplicate. After the run, data were analyzed by using Xponent software (1.9 version, Luminex 200 System; Thermo Fisher Scientific) and the final concentration of each cytokine was expressed in pg/mL.

### 2.13. Histochemistry and Immunofluorescence

Tissue sections, 5 *μ*m in thickness, were used for histology. By immunofluorescence, injected cells were detected by GFP antibody (Abcam, Cambridge, UK) [32]. Cycling epithelial and smooth muscle cells (SMCs) were identified by Ki67 labeling (Vector Laboratories, Burlingame, CA, USA). The mucous cell metaplasia was assessed by the immunolabeling with anti-mucin 5AC antibody (Abcam). Mucin-positive cells were quantified in the epithelial layer of the bronchi by counting labeled cells per total number of cells within the airway epithelium. Inflammatory cells were recognized by CD45 expression (Novus Biologicals, Littleton, CO, USA). Macrophages were identified by pan-macrophage marker F4/80 (Novus Biologicals) and M1/M2 subsets by inducible nitric oxide synthase (iNOS, Thermo Fisher Scientific) and arginase-1 expression (Abcam), respectively. Terminal deoxynucleotidyltransferase-mediated dUTP nick end labeling (TUNEL) assay was used for the detection of apoptosis in the lung tissue, according to manufacturer's instructions (Clontech Laboratories, Mountain View, CA, USA). Apoptotic index was expressed as the number of TUNEL^+^ cells per million of cells within inflammatory infiltrate. Nuclei were stained with DAPI (Sigma-Aldrich). Fluorescein isothiocyanate (FITC) and tetramethylrhodamine-5-(and 6)-isothiocyanate (TRITC) conjugated secondary antibodies were used (Jackson ImmunoResearch, Newmarket, UK). For the assessment of inflammation, tissue sections were stained with H&E (Sigma-Aldrich). The number of mast cells per mm^2^ of the lung tissue was measured after staining with toluidine blue (Sigma-Aldrich) [33]. Sample area was measured with Image Pro Plus software (Media Cybernetics, Rockville, MD, USA). Samples were analyzed with Leica DM 5000B microscope (Leica Microsystems, Wetzlar, Germany) and Zeiss LSM 700 confocal microscope (Zeiss, Oberkochen, Germany).

### 2.14. PCR for Detection of GFP DNA

For PCR detection of GFP, paraffin sections were obtained from the lungs of mice in which GFP^+^ cells were previously detected by immunohistochemistry. Tissue sections were deparaffinized and 100 ng genomic DNA, extracted with the QIAamp DNA kit (Qiagen), were mixed with GFP primers. Cycling conditions were as follows: 94°C for 30 sec, followed by 30 cycles of amplification (94°C for 30 sec, 62°C for 30 sec, and 72°C for 30 sec), with a final incubation at 72°C for 3 min. PCR products were run onto agarose gel for the detection of the GFP band (amplicon size: 315 bp). DNA extracted from GFP rats was used as positive control while tissue sections from medium-injected mice were used as negative control [34].

### 2.15. Statistical Analysis

Results were reported as mean ± SD. Data were analyzed by using GraphPad Prism (GraphPad Software, San Diego, CA, USA). Significance between two comparisons was determined by Student's* t*-test and among multiple comparisons by one-way ANOVA and Bonferroni's posttest. Lung reactivity curves were compared using a two-way ANOVA followed by Bonferroni's posttest. A value of *P* < 0.05 was considered as significant.

### 2.16. Animal Studies Approval

The experimental protocol was approved by the Animal Care and Use Committee of the Second University of Naples (1966/7.17.2012). Animal care complied with Italian regulations on protection of animals used for experimental and other scientific purposes (116/1992) as well as with the EU guidelines for the use of experimental animals (2010/63/EU). Mice were housed in the Animal Facility of the Second University of Naples. Food and water were supplied ad libitum. Room temperature was kept at 22°C–24°C and relative humidity at 40%–50%. The day/night cycle was set at 12 h/12 h. Mice were acclimatized for one week before starting any procedures and, during this time, they were submitted to a daily handling to get them used to manipulation, thus reducing experimental variability due to the stress due to the procedures. All treatments were performed by experienced operators and in asepsis. During challenge (aerosol), freely moving mice were kept in a suitable chamber with the minimal stress. No animal became severely ill or died during the in vivo procedures. Mice subjected to lung reactivity were exsanguinated after incision of the renal vein, while the remaining animals were sacrificed by cervical dislocation.

## 3. Results

### 3.1. Identification and Isolation of c-kit^+^ Cells

**Figure 1:**
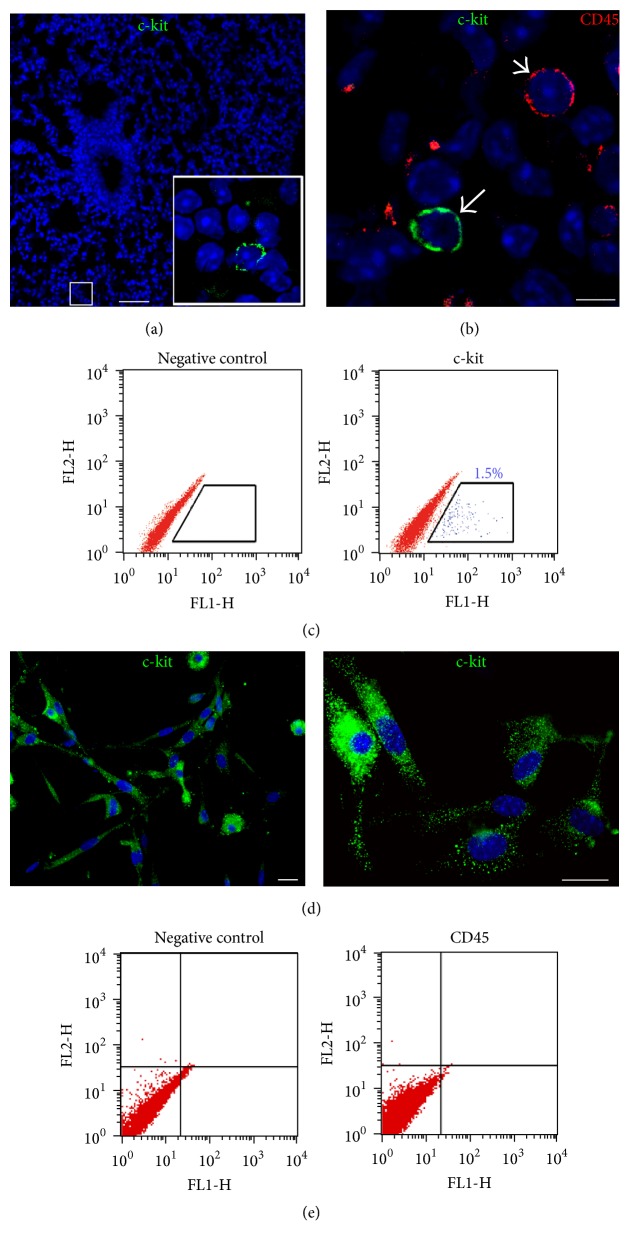
In situ detection, isolation, and characterization of c-kit^+^ cells. (a) c-kit^+^ cells (green, inset) in a normal adult mouse lung. (b) An example of c-kit^+^ cell (green, arrow) and CD45^+^ cell (red, arrowhead). (c) FACS scatter plots showing the expression of c-kit in a population of small lung cells. (d) Expression of c-kit (green) on sorted cells shown by immunocytochemistry. (e) FACS analysis of c-kit sorted cells showing lack of expression of hematopoietic lineage marker CD45. Scale bars, (a) 50 *μ*m, (b) 5 *μ*m, and (d) 20 *μ*m.

c-kit^+^ cells were detected in adult mouse lung tissue (Figure 1(a)). They did not express CD45, excluding hematopoietic lineage (Figure 1(b)), and were negative for characteristic epithelial markers, such as pan-cytokeratin, thyroid transcription factor 1, and cystic fibrosis transmembrane conductance regulator as well as mast cell tryptase (not shown). FACS analysis showed a small fraction of c-kit^+^ cells in physically separated small lung cells obtained by tissue enzymatic digestion (Figure 1(c)). Afterward, immunosorting yielded c-kit-enriched cell population that was negative CD45 (Figures 1(d) and 1(e)).

### 3.2. Airway Hyperresponsiveness and Engraftment of c-kit^+^ Cells

**Figure 2:**
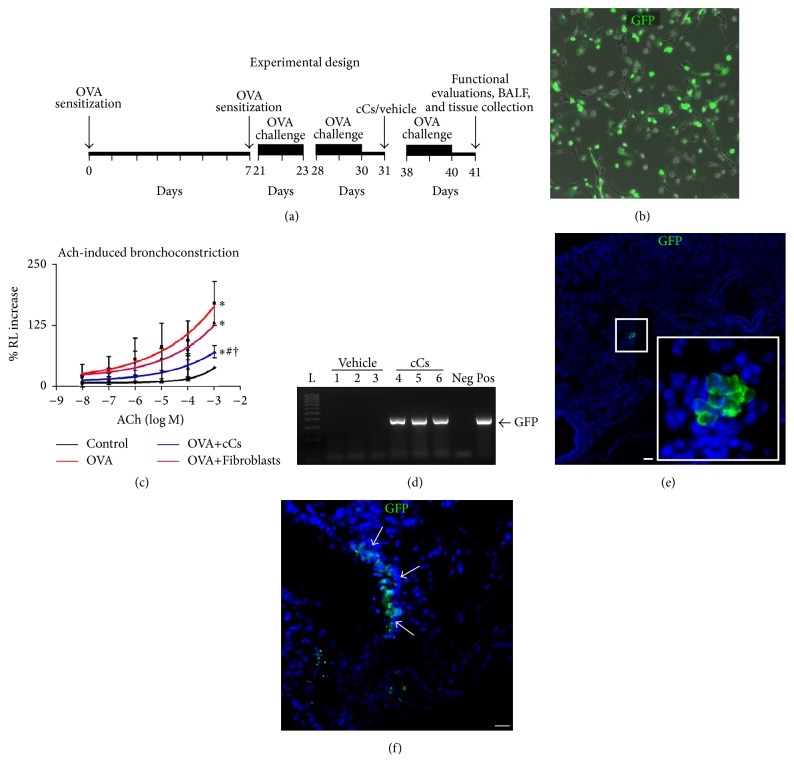
In vivo engraftment of GFP-labeled c-kit^+^ cells and pulmonary function. (a) Scheme of in vivo experiments. (b) Efficiency of GFP transfection. Phase contrast and GFP fluorescence image of living c-kit^+^ cells after lentiviral transduction. (c) Airway reactivity to acetylcholine (ACh, in moles) as change in resistance (RL) in control, asthmatic (OVA), c-kit^+^ cell-treated (OVA+cCs), and fibroblast-treated (OVA+Fibroblasts) mice. (d) Detection of GFP gene by PCR in the lungs of vehicle- and c-kit^+^ cell-treated mice. (e, f) Engraftment of GFP-labeled cells after intratracheal administration. Scale bars, (e, f) 20 *μ*m. ^*∗*^*P* < 0.05 versus control; ^#^*P* < 0.05 versus OVA; ^†^*P* < 0.05 versus OVA+Fibroblasts.

c-kit^+^ cells were intratracheally instilled in experimental animals in which airway hyperresponsiveness was induced by sensitization and challenge with OVA (Figure 2(a)). Prior to administration, the cells were transduced with GFP to trace them within the recipient tissue (Figure 2(b)). In OVA mice, ACh-induced bronchoconstriction increased with respect to controls, while a significant reduction in bronchial hyperreactivity was observed in the animals that received c-kit^+^ cells. Lung fibroblasts did not produce a significant beneficial effect (Figure 2(c)). This comparative analysis using an additional cell type (i.e., lung fibroblasts) was done to elucidate whether the modulation of lung function was dependent on the cell class. The presence of GFP^+^ cells in the recipient tissue was evidenced by PCR for GFP gene and immunohistochemistry that showed GFP-tagged cells at the level of bronchial epithelium and lung parenchyma (Figures 2(d)–2(f)).

### 3.3. Airway Remodeling

**Figure 3:**
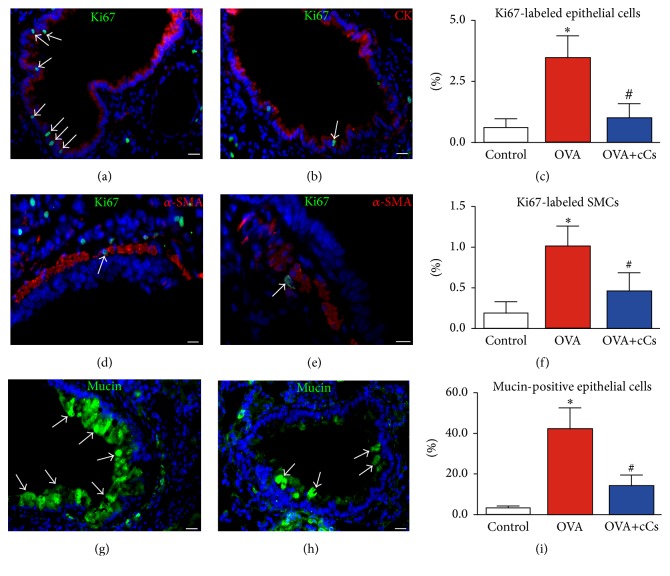
c-kit^+^ cells positively interfere with the remodeling of asthmatic airways. (a–c) Proliferating (Ki67, green, arrows) epithelial cells (pan-cytokeratin, CK, red) in vehicle- (a) and c-kit^+^ cell-treated (b) animals. (c) The fraction of Ki67^+^ epithelial cells. (d-e) Cycling (Ki67, green, arrows) SMCs (*α*-SMA, red) in the airway wall of vehicle- (d) and c-kit^+^ cell-treated (e) mice. (f) The fraction of Ki67^+^ SMCs. (g-h) Mucin-positive goblet cells (green, arrows) in epithelium of vehicle- (g) and c-kit^+^ cell-treated (h) asthmatic mice. (i) The percentage of epithelial cells expressing mucin. Data in control, OVA, and OVA+cCs mice are the mean ± SD. Scale bars, (a, b, d, e) 20 *μ*m and (g, h) 10 *μ*m. ^*∗*^*P* < 0.05 versus control; ^#^*P* < 0.05 versus OVA. SMCs: smooth muscle cells; *α*-SMA: *α*-smooth muscle actin.

Proliferating epithelial and SMCs were detected in the airways documenting the contribution of hyperplasia to the airway remodeling. In OVA mice, the fraction of Ki67^+^ epithelial and SMCs was nearly 6-fold and 5-fold higher than that in controls. However, the proliferation index was reduced by 71% in epithelium and by 54% in smooth muscle layer after c-kit^+^ cells administration (Figures 3(a)–3(f)). The intense mucus metaplasia observed in OVA mice was significantly reduced by c-kit^+^ cells (Figures 3(g)–3(i)). These results document that c-kit^+^ cells are able to attenuate the hyperplastic phase of both bronchial epithelium and smooth muscle mass, thus reducing airway remodeling.

### 3.4. Inflammation and Immunomodulation

**Figure 4:**
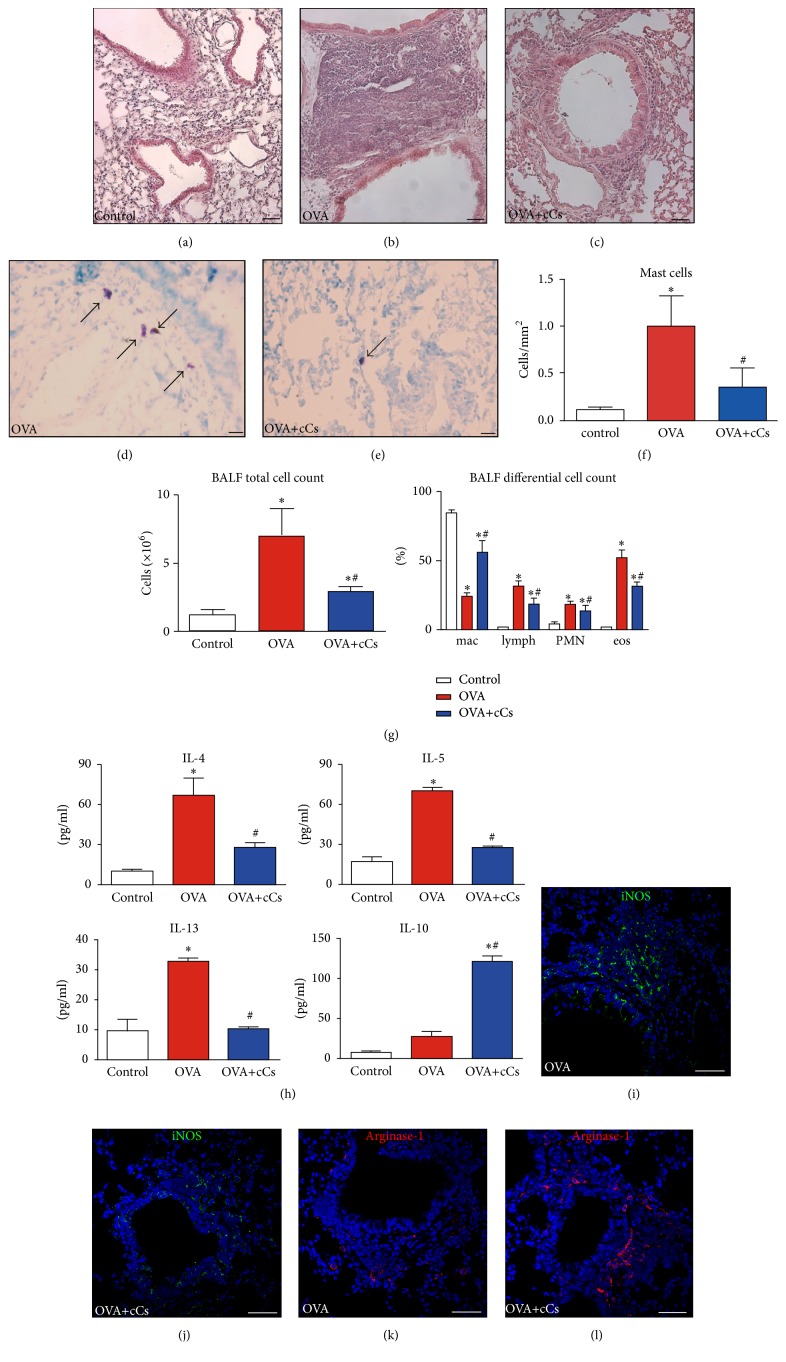
c-kit^+^ cells interfere with lung inflammation. (a–c) With respect to controls (a), massive accumulation of inflammatory cells is visible in the lungs of OVA animals (b). The infiltration is markedly reduced in c-kit^+^-treated asthmatic mice (c). (d, e) Mast cells (toluidine blue, arrows) in the lungs of vehicle- (d) and c-kit^+^ cell-treated (e) asthmatic mice. (f) The number of mast cells per mm^2^ of tissue. (g) Total cell number and differential cell count in the BAL collected from control, OVA, and OVA+cCs mice. (h) Cytokine levels measured in the BAL. (i–l) Macrophage polarization as revealed by iNOS (green) and arginase-1 (red) immunostainings in OVA and OVA+cCs lungs. Scale bars, (a–c, i–l) 50 *μ*m and (d, e) 20 *μ*m. Data in control, OVA, and OVA+cCs mice are the mean ± SD. ^*∗*^*P* < 0.05 versus control; ^#^*P* < 0.05 versus OVA. BALF: bronchoalveolar lavage fluid; mac: macrophages; lymph: lymphocytes; PMN: polymorphonuclear leukocytes; eos: eosinophiles; iNOS: inducible nitric oxide synthase.

The increase in peribronchial infiltration of the inflammatory cells was evident in the OVA group. However, c-kit^+^ cells determined a significant reduction in the extent of inflammatory infiltration (Figures 4(a)–4(c)). Additionally, the increased number of mast cells observed in OVA animals became markedly lowered after cell administration (Figures 4(d)–4(f)). The analysis of BAL indicated an ongoing inflammation in OVA mice, given the increase in the total cell number and in the proportion of lymphocytes, polymorphonucleated cells, and eosinophils. On the contrary, total and differential cell counts evidenced a less prominent grade of inflammation in cell-treated animals (Figure 4(g)). Interestingly, the augmented fraction of macrophages, reported also with MSCs [35], suggests the involvement of macrophage activation in the anti-inflammatory response. Moreover, BAL from OVA mice showed elevated concentrations of Th2 proinflammatory cytokines (i.e., IL-4, IL-5, and IL-13) while the levels of IL-10 were unchanged. c-kit^+^ cells administration determined a reduction in the levels of Th2 proinflammatory cytokines and an increase of IL-10 (Figure 4(h)). Since macrophages play critical role in inflammatory modulation, their recruitment and polarization have been determined. The instillation of c-kit^+^ cells did not affect the total number of tissue macrophages (marked by pan-marker F4/F80) in the OVA-treated lungs (not shown). However, in cell-treated animals, the induction of arginase-1^+^ M2-like macrophages activation marker was more prominent, while iNOS^+^ M1 phenotype was less represented (Figures 4(i)–4(l)) suggesting preferential polarization into IL-10-producing M2-like cells.

These data indicate that the intratracheal administration of c-kit^+^ cells can be responsible for the control of the local inflammatory process.

**Figure 5:**
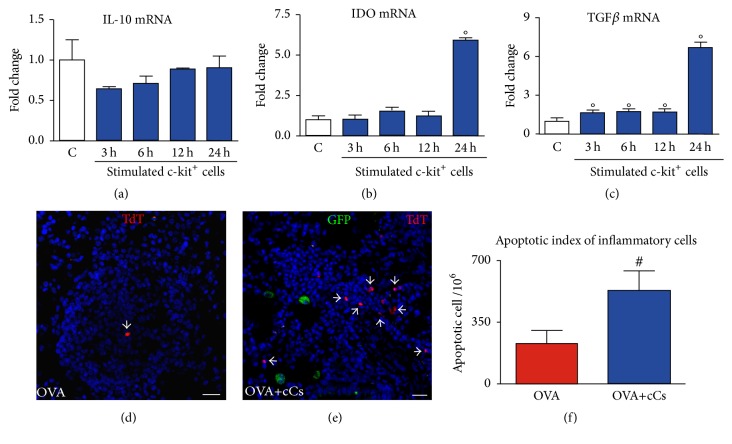
Immunomodulatory properties of c-kit^+^ cells. (a–c) mRNA expression of IL-10 (a), IDO (b), and TGF*β* (c) measured by RT-PCR in unstimulated (C, white bars) and stimulated (blue bars) c-kit^+^ ± SD. °*P* < 0.05 versus unstimulated cells. (d, e) Apoptotic inflammatory cells (TdT, red, arrowheads) within inflammatory infiltrates in the lungs of vehicle- (d) and c-kit^+^ cell-treated (e) asthmatic mice. GFP^+^ cells (green) are present in the close proximity of apoptotic inflammatory cells in c-kit^+^ cell-treated asthmatic mouse. (f) Apoptotic index of inflammatory cells in OVA and OVA+cCs animals. ^#^*P* < 0.05 versus OVA mice. Scale bars, (d, e) 20 *μ*m. IDO: indoleamine 2,3-dioxygenase; TGF*β*: transforming growth factor *β*; TdT: terminal deoxynucleotidyltransferase-mediated dUTP nick end labeling.

In the search for potential mechanisms responsible for positive effects of c-kit^+^ cells on the inflammation, isolated c-kit^+^ cells were stimulated in vitro with IFN*γ* and TNF*α*. Real-time PCR showed the presence of IL-10 transcript in resting c-kit^+^ cells, but no difference was revealed after proinflammatory stimulation (Figure 5(a)). Interestingly, when stimulated, c-kit^+^ cells showed increased levels of mRNA for IDO and TGF*β* (Figures 5(b) and 5(c)). TGF*β*-dependent pathways are essential for IDO regulatory responses [36], and IDO-producing cells can promote death of lymphocytes by modifying the local microenvironment [37]. In this regard, apoptotic rate within inflammatory infiltrates was measured and became more than 2-fold higher in cell-treated animals (Figures 5(d)–5(f)). The presence of GFP^+^ cells in the proximity of the apoptotic inflammatory cells suggests that c-kit^+^ cells may downregulate, in a paracrine manner, the extent of the inflammatory infiltrate.

## 4. Discussion

Asthma is a common chronic disorder of the airways characterized by predominant eosinophilic inflammatory pattern associated with the whole spectrum of asthma severity, from mild-to-moderate to severe uncontrolled disease [38, 39]. Its pathophysiology involves intermittent airway obstruction, bronchial hyperreactivity, chronic eosinophilic airway inflammation, goblet cell hyperplasia, and airway structural changes in response to a chronic Th2 immune response [40]. Allergens and some diseases have been implicated in the exacerbation of asthma in both animal models and humans [41–43].

Increasing reports have indicated that, among several lung resident stem cell types, c-kit^+^ cells are important for pulmonary homeostasis and repair [16–19, 23, 44], but, to date, their capacity to regulate inflammation has never been studied. In the present work, mouse c-kit^+^ cells were identified within the normal mouse lung and located mostly throughout the lung parenchyma and occasionally in the epithelial layer. In order to observe their behavior in an experimental model of lung disease, we performed an exogenous cell delivery. Considering the small number present in a mouse lung, cells were administered intratracheally after isolation and in vitro expansion. The intratracheal route for cell delivery used in our study is a methodology that has several advantages. This type of local administration, rarely carried out, provides benefits that cannot be extended to a systemic infusion, such as the reduction of cells number to be infused and the low risk to engraft other organs.

In our model, the proliferation rate of the epithelium, the fraction of cycling SMCs, and activation of goblet cells were significantly higher in the OVA group. This is consistent with the notion that bronchial epithelial hyperplasia, smooth muscle layer thickening, and increased number of mucus-producing cells are key components of airway remodeling occurring in allergic asthma [2, 45]. Interestingly, mice that received c-kit^+^ cells exhibited lowered proliferation index of both bronchial epithelium and airway SMCs together with the decrease in the number of goblet cells. Importantly, the positive effects on the remodeling were coupled with the improved functional properties of the airways, manifested as reduced hyperreactivity to ACh. These findings demonstrate that c-kit^+^ cells, when placed in this specific pathological inflammatory condition, can interfere with the cellular aspect of airway remodeling that contributes to the evolution of the disease. In our experimental setting, administration of lung fibroblast did not improve AHR. This indicates that the modulation of lung function cannot be solely ascribed to the instillation of a “biomass” into the tissue but represents a cell type specific phenomenon. Our data do not exclude the possibility that other lung stem/progenitor cells may be involved in the response to injury. Understanding the potential functional relationship between different cell populations in the diseased lung remains an open and challenging question.

The attenuation of the immune and inflammatory processes can significantly affect bronchial function and homeostasis. Indeed, MSCs have been shown to be effective mostly because of their anti-inflammatory properties while their capacity to differentiate into epithelial cells remains to be clarified [20, 46, 47]. Similarly, in our study the inflammatory processes, typical of this OVA-induced model of experimental AHR, were significantly influenced by exogenously administered c-kit^+^ cells. The observed decrease in the number of inflammatory cells in the BAL and the reduced infiltration of lung tissue suggested the possible immunomodulatory properties of tested cell type. The beneficial effects were associated with a marked reduction of proinflammatory cytokines IL-4, IL-5, and IL-13 in BAL. In contrast, IL-10, a potent inhibitor of several proinflammatory cytokines [48–50], was significantly increased. c-kit^+^ cells were able to produce IL-10 in vitro but there was no difference in the IL-10 transcript after exposure to canonical proinflammatory stimuli. This indicates that the increase in IL-10 in BAL of cell-injected animals was probably due to the capacity of c-kit^+^ cells to stimulate other cells to secrete this pleiotropic immunosuppressive cytokine [51, 52]. In this regard, the involvement of macrophages that represent a potent source of IL-10 was considered. Activated macrophages regulate bronchial inflammation and AHR in asthma. Whereas the classically activated macrophages (M1 subtype) respond to inflammatory stimuli producing high levels of proinflammatory cytokines, M2-like macrophages secrete a range of anti-inflammatory factors, such as IL-10 [53–55]. Our data demonstrated that administration of c-kit^+^ cells determined a polarization toward M2-like subtype, suggesting that regulatory macrophages, among other cell classes able to produce and release IL-10, may be in part responsible for the inhibition of inflammation, thus mediating the positive effects of c-kit^+^ cells on AHR and eosinophilic accumulation.

Furthermore, IDO and TGF*β* mRNAs were markedly increased in stimulated cells. IDO modulates the immune response by inducing a rapid depletion of the amino acid tryptophan and by leading to the formation of proapoptotic metabolites [37], whereas TGF*β* is able to downregulate the allergic response [56]. Moreover, IDO, TGF*β*, and prostaglandin E2 secreted by adipose-derived MSCs ameliorate airway inflammation and improve lung function [57]. The observations that GFP-labeled c-kit^+^ cells were present within inflammatory infiltrates and that the apoptosis of inflammatory cells was higher in cell-treated animals support the hypothesis of local immunoregulation as a possible regulatory mechanism.

Several factors have been proposed to mediate immunosuppressive effect, including IDO, TGF*β*, prostaglandin E2, hepatocyte growth factor, nitric oxide, and IL-10. A number of specific immune cell populations are involved and their recruitment and direct or indirect capacity to secrete biological factors may explain paracrine immunosuppressive activity observed in experimental studies with stem cells or trophic molecules. Myeloid-derived suppressor cells (MDSCs) are innate immune cells characterized by their potential to control T cell responses and inhibit inflammation. It has been reported that MDSC accumulation positively correlates with the increase of IL-10 by both direct production and an interaction between MDSCs and macrophages [58, 59]. There is also evidence for distinct roles of conventional and plasmacytoid dendritic cells in regulating T cell-mediated adaptive immunity in the lung [60]. Conventional dendritic cells promote Th2 sensitization [61], whereas plasmacytoid dendritic cells exert anti-inflammatory effects for their capacity to release IDO thus inhibiting T cell responses [62, 63]. To date, no study addressed the in vivo influence of c-kit^+^ cells on immune system, and the only relevant information can be extrapolated from paracrine effects of MSCs. MSCs regulate, via TGF*β* and/or IDO, the function of CD4^+^ CD25^+^ FoxP3^+^ regulatory T cells and NK cells and, through prostaglandin E2, the maturation/activation of dendritic cells [64, 65]. However, in the search of mechanisms by which MSCs mediate immunomodulation, cell-to-cell contact should be considered as well, as demonstrated by in vitro studies showing a direct participation of MSCs in the regulation of immune cell behavior [66, 67]. We did not examine the effect of c-kit^+^ cells on different populations of lymphocytes; the data on macrophage polarization suggest that c-kit^+^ cells can promote accumulation/activation of cells releasing immunosuppressive cytokines. Our data regarding the improved airway function and the wide range of actions that c-kit^+^ cells share with MSCs make the possible interaction with a variety of immune cells more than reasonable. Further investigations and ad hoc experiments are needed to clarify the mechanisms involved in the modulation of anti-inflammatory response driven by c-kit^+^ cells.

## 5. Conclusion

In the present study we report that c-kit^+^ cells, isolated from adult lung, improve lung function through the restraint of inflammatory processes. The secretion of multiple soluble mediators, switching off a proinflammatory state and favoring an anti-inflammatory condition, can explain the immunosuppressive effect of c-kit^+^ cells in vivo, which resembles the known action attributed to MSCs. The capability to modify the inflammatory environment, possibly in a paracrine and cell-to-cell contact manner, can be considered as a new feature of c-kit^+^ cells, in addition to their participation in tissue homeostasis [16–18].
